# Draft Genome Sequence of Escherichia coli UMB9246, Isolated from the Bladder of a Woman with Recurrent Urinary Tract Infection

**DOI:** 10.1128/MRA.00437-20

**Published:** 2020-06-04

**Authors:** Mikalina Belmonte, Taylor Miller-Ensminger, Adelina Voukadinova, Alan J. Wolfe, Catherine Putonti

**Affiliations:** a Department of Biology, Loyola University Chicago, Chicago, Illinois, USA; b Bioinformatics Program, Loyola University Chicago, Chicago, Illinois, USA; c Department of Microbiology and Immunology, Stritch School of Medicine, Loyola University Chicago, Maywood, Illinois, USA; d Department of Computer Science, Loyola University Chicago, Chicago, Illinois, USA; University of Maryland School of Medicine

## Abstract

Escherichia coli is a Gram-negative, motile, rod-shaped bacterium that causes the majority of uncomplicated urinary tract infections (UTIs). Here, we report the draft genome of E. coli strain UMB9246, an isolate from a woman with recurrent UTI.

## ANNOUNCEMENT

Pathogenic strains of Escherichia coli can cause intestinal, urinary tract, and internal infections, and E. coli isolates from multiple body sites have been sequenced (1). Specifically, uropathogenic E. coli (UPEC) strains are responsible for the majority of infrequent urinary tract infections (UTIs) in humans (2, 3). Recurrence of UTIs is frequent (4). Recurrent UTIs (rUTIs) are considerably more complicated and nuanced than infrequent UTIs. Here, we isolated E. coli strain UMB9246 from a catheterized urine sample obtained from a woman with an rUTI.

E. coli strain UMB9246 was collected as part of a prior institutional review board (IRB)-approved study (University of California, San Diego, IRB no. 170077AW) using the expanded quantitative urine culture (EQUC) protocol (5). Briefly, 100 μl of urine was spread onto a 5% sheep blood agar plate and incubated overnight at 35°C with 5% CO_2_ for 24 h. Each morphologically distinct colony was then further purified through successive plating. The genus and species for this isolate were determined via matrix-assisted laser desorption ionization–time of flight (MALDI-TOF) mass spectrometry as previously described (5). The isolate was then stored at −80°C until sequencing. From this freezer stock, the sample was streaked onto a Columbia nalidixic acid (CNA) agar plate and incubated at 35°C with 5% CO_2_ for 24 h. A single colony was selected and incubated in LB broth at 37°C with shaking for 24 h. DNA was extracted using the DNeasy blood and tissue kit following the manufacturer’s protocol for Gram-positive bacteria with the following alterations. We used 230 μl of lysis buffer (180 μl of 20 mM Tris-Cl, 2 mM sodium EDTA, and 1.2% Triton X-100 and 50 μl of lysozyme) in step 2, and in step 5 of the protocol, we incubated the sample at 56°C for 10 min. DNA was quantified using the Qubit fluorometer and sent to the Microbial Genome Sequencing Center (MiGS) at the University of Pittsburgh for sequencing. The DNA was enzymatically fragmented using an Illumina tagmentation enzyme, and indices were attached using PCR. The DNA was sequenced using an Illumina NextSeq 550 flow cell, producing 1,408,737 pairs of 150-bp reads. The raw reads were trimmed using Sickle v1.33 (https://github.com/najoshi/sickle) and assembled using SPAdes v3.13.0 with the “only-assembler” option for k values of 55, 77, 99, and 127 (6). The genome coverage is 71× and was calculated using BBMap v38.47 (https://sourceforge.net/projects/bbmap/). The genome assembly was first annotated using PATRIC v3.6.3 (7). The NCBI Prokaryotic Genome Annotation Pipeline (PGAP) v4.11 (8) was used to annotate the publicly available genome. The genome assembly was also examined using PHASTER (9) and CRISPRCasFinder (10) to identify prophage sequences and CRISPR spacer arrays, respectively. Default parameters were used for each software tool, unless previously stated.

The E. coli UMB9246 draft genome is 5,057,732 bp long in 90 contigs with an *N*_50_ score of 159,145 bp and a GC content of 50.8%. The PGAP annotation identified 4,691 protein-coding genes, 5 rRNA operons, and 77 tRNAs. PHASTER predicted 1 questionable, 2 incomplete, and 1 intact phage for this strain. CRISPRCasFinder found 2 CRISPR arrays. PATRIC identified in the genome assembly both virulence factors and antibiotic resistance genes, including those for tetracycline resistance. Further analysis of this strain and other isolates from individuals suffering from uncomplicated UTIs and rUTIs will give us insight into the mechanisms for recurrence.

### Data availability.

This whole-genome shotgun project has been deposited in GenBank under the accession no. JAAUVS000000000. The version described in this paper is the first version, JAAUVS010000000. The raw sequencing reads have been deposited in SRA under the accession no. SRR11441020.
